# Beyond fluid overload: uncovering the hidden causes of dyspnea in hemodialysis patients

**DOI:** 10.3389/fmed.2026.1809791

**Published:** 2026-05-28

**Authors:** Kristina Buryskova Salajova, Jan Malik, Eva Strakova, Zuzana Hladinova, Zdenka Hruskova, Oskar Zakiyanov, Tomas Parviz Mirchi, Simona Janakova, Vladimir Tesar, Pavel Michalek, Kristyna Michalickova, Anna Valerianova

**Affiliations:** 1 Third Department of Internal Medicine, General University Hospital, First Faculty of Medicine, Charles University, Prague, Czechia; 2 Department of Nephrology, General University Hospital, First Faculty of Medicine, Charles University, Prague, Czechia; 3 Department of Anesthesiology and Intensive Care, General University Hospital, First Faculty of Medicine, Charles University, Prague, Czechia

**Keywords:** Diffusing capacity for carbon monoxide, dyspnea, end-stage kidney disease, heart failure, hemodialysis, hydration, lung function, muscle strength

## Abstract

**Background:**

Shortness of breath is a common and complex symptom in patients with advanced kidney failure treated with hemodialysis. It may result from excess fluid accumulation, cardiac dysfunction, or impaired lung performance. We studied the interactions between hydration status, pulmonary function, and muscle strength.

**Methods:**

This cohort study included 29 patients on maintenance hemodialysis. Each participant underwent lung function testing, diffusing capacity for carbon monoxide measurement, handgrip strength assessment, body composition analysis by bioimpedance, echocardiography, and laboratory testing. All examinations were performed within 1 h before and after the same hemodialysis session.

**Results:**

Hemodialysis significantly reduced total and extracellular body water and increased total lung capacity (5.11 ± 1.18 vs. 5.34 ± 1.27; *p* = 0.005), forced expiratory volume in 1 s (2.44 ± 0.88 vs. 2.54 ± 0.9 L; *p* = 0.022), and diffusing capacity for carbon monoxide [14.48 (4.35) vs. 15.73 (4.78) mL/min/mmHg; *p* = 0.0014]. Muscle strength decreased slightly after hemodialysis (25,31 ± 12,82 vs. 23,79 ± 12,07 kg; *p* = 0,008), but this change was not correlated with the improvements in lung function. An improvement in gas transfer was associated with an increase in heart rate. Increased left ventricular filling pressure was linked to reduced pre-dialysis lung volume.

**Conclusions:**

In our study, single hemodialysis session led to rapid improvements in lung volume and gas exchange, mainly due to reduced body fluid, as well as associated hemodynamic changes, rather than changes in muscle strength. Therefore, this study further underscores the need of a careful fluid management not only for maintaining respiratory performance but also overall wellbeing in patients on hemodialysis. Further studies with larger cohorts and long-term follow-up are needed to confirm these findings.

## 1 Introduction

The prevalence of chronic kidney disease (CKD) has been increasing worldwide, primarily because of the rising incidence of metabolic syndrome, particularly hypertension and diabetes mellitus. CKD affects approximately 13% of the global population (1), and the prevalence of end-stage kidney disease (ESKD) in developed countries is approximately 1,210 per million people (2, 3).

Dyspnea is among the most common symptoms of ESKD and often results from pulmonary congestion, cardiac failure, anemia, or other comorbidities. Overhydration, a frequent problem in hemodialysis patients, can contribute to dyspnea and can be assessed by clinical examination, bioimpedance, or ultrasound (4, 5). Dyspnea not only worsens quality of life but is also associated with poor outcomes (6). Interdialytic weight gain (IDWG), which is caused by fluid and sodium retention, can exacerbate lung congestion, although a higher IDWG may also indicate better nutrition and muscle strength (7, 8). Furthermore, rapid osmotic and ionic shifts during dialysis affect muscle performance (9, 10). However, the relationships among lung function, muscle strength, and body composition in this population remain unclear.

Given its multifactorial nature, ESKD-related dyspnea poses diagnostic and therapeutic challenges. Pulmonary function tests, including spirometry and diffusing capacity for carbon monoxide (DLCO), provide insight into ventilatory capacity and alveolar–capillary gas transfer (11, 12). DLCO is influenced by pulmonary congestion, pleural effusion, and interstitial or emphysematous changes (13). Adequate respiratory muscle strength is essential for normal ventilation but is frequently reduced in hemodialysis patients (14, 15), which contributes to impaired lung function and increased mortality (16–18).

Altogether, multiple mechanisms contribute to dyspnea in ESKD patients, and their interrelationships are not yet fully understood. Therefore, we analyzed the effects of a single hemodialysis session in ESKD patients using a combination of functional pulmonary testing, DLCO, echocardiography, bioimpedance analysis, dynamometry and basic laboratory tests. Specifically, the aims of this cohort study were as follows:

1. To describe the relationships between pulmonary function testing and DLCO with hydration status, 2. To reveal the impact of a single hemodialysis on muscle strength and its relationship with pulmonary function, and 3. To analyze the relationships between pulmonary function, selected laboratory tests and structural cardiac changes.

## 2 Materials and methods

We screened all patients who attended our hemodialysis unit. The inclusion criteria were as follows: willingness to participate, age over 18 years, and chronic hemodialysis via arteriovenous access or a permanent catheter. The exclusion criteria were acute decompensation of chronic cardiac failure, acute decompensation of chronic lung disease, acute respiratory infection, and inability or refusal to cooperate. All patients provided written informed consent, and the study complied with the principles of the Declaration of Helsinki. All examinations (details below) were performed within 1 h before and then within 1 h after the same hemodialysis session. Bicarbonate hemodialysis was used in all patients.

### 2.1 Lung function testing

Spirometric examination and measurement of carbon monoxide diffusing capacity of the lungs were performed under standard conditions according to the national guidelines (19) and recorded using an EasyOne Pro^®^ LAB spirometer (NDD Medical Technologies, Switzerland). The examination was performed by one physician (KBS) and one nurse (ES). Lung volumes and flow volumes, including slow vital capacity (SVC), forced vital capacity (FVC), total lung capacity (TLC), forced expiratory volume in the 1 s (FEV 1), forced expiratory flow at 25–75% of exhaled effortful vital capacity (FEF 25–75%) and the FEV1/FVC ratio along with DLCO, were analyzed along with the assessment of the recorded curves. DLCO was corrected to the actual hemoglobin level and is expressed in units of mL/min/mmHg and it represents the alveolar–capillary membrane function.

### 2.2 Muscle strength examination

Handgrip strength was used as a surrogate marker of respiratory muscle strength. Previous studies have demonstrated significant correlations between handgrip strength and respiratory muscle strength parameters, suggesting that peripheral muscle strength reflects global muscle function, including respiratory muscles (20, 21). Dynamometry was performed with a hydraulic hand dynamometer (Saehan, Korea) to assess maximal muscle strength in the hand without fistula or, in patients without a fistula, in the dominant hand. Hand grip strength was measured three times, and the highest value was recorded.

### 2.3 Hydration assessment

Bioelectrical impedance analysis (BIA) using a body composition monitor (FMC, Germany) was used to estimate hydration and measure other body composition parameters (hand to foot). The measurement was performed on the opposite side of the hemodialysis access. BIA was used to measure the level of overhydration in liters, but this value is not adjusted to body size. Therefore, we also used the relative hydration index (overhydration adjusted to extracellular water) as a more accurate assessment of hydration status (22).

### 2.4 Dyspnea evaluation

Dyspnea was assessed using the New York Heart Association (NYHA) functional classification. Each patient was assigned to one of four classes (I–IV) based on the severity of symptoms and their impact on daily physical activity. In addition, patients were asked standardized questions focusing on the presence and intensity of breathlessness at rest and during exertion, changes in dyspnea between dialysis sessions, and limitations in routine activities due to shortness of breath. The assessment was performed within 1 h before and repeated within 1 h after the same hemodialysis session by the same examiner to ensure consistency.

### 2.5 Expert echocardiography

Expert echocardiography was performed once, no longer than 2 months before lung function testing and was performed at least 24 h after the previous hemodialysis and no more than 24 h before the subsequent session. Therefore, echocardiographic measurements were obtained under relatively stable volume conditions rather than during periods of rapid fluid change. The examination was performed using a matrix echocardiography probe of a Vivid E95 device (General Electric, Vingmed, Norway). We performed a detailed analysis of the cardiac chamber volumes, quantification of valvular disease and calculation of cardiac output (CO) using the left ventricular outflow tract diameter and velocity–time interval. Left ventricular filling pressure was calculated from the mitral annular velocity and mitral flow velocity according to the guidelines (23). Examinations were performed by one of three examiners experienced in cardionephrology (K.B.S., A.V. and J.M.). Interindividual variability in cardiac chamber diameter was 5%, and variability chamber volume was 8%.

### 2.6 Laboratory tests

Laboratory parameters were analyzed from blood samples obtained during the hemodialysis session to minimize procedural invasiveness. Blood samples were consistently drawn from the arterial line of the dialysis circuit at the very beginning of the hemodialysis session, prior to significant ultrafiltration and dialysate exchange and at the end of the hemodialysis session. The analyzed parameters included routine biochemical tests, complete blood count, and blood gas analysis.

### 2.7 Statistical analysis

The statistical software STATISTICA (StatSoft Inc., USA) was used for statistical analysis. Variables were tested using the Shapiro–Wilk test to assess data distribution (Gaussian vs. non-Gaussian). For data with a non-Gaussian distribution, the Wilcoxon matched pairs signed rank test was used, whereas a paired *t*-test was used for data with a Gaussian distribution. Results are expressed as either the mean ± standard deviation for the normal data distribution and as median (interquartile range) for the non-Gaussian distribution. Spearman's rank correlation and regression analyses were performed to evaluate the association between variable differences related to hemodialysis. Given the exploratory nature of the study and limited sample size, no correction for multiple comparisons was applied; thus, findings should be considered hypothesis-generating.

Based on the observed paired differences, the effect size for DLCO was large (Cohen's d = 0.73), corresponding to an estimated statistical power of approximately 90% with a sample size of 29 patients. Similarly, TLC demonstrated a moderate effect size (d = 0.59), providing approximately 80% power. In contrast, other parameters showed smaller effect sizes and lower statistical power, and should therefore be interpreted as exploratory.

## 3 Results

### 3.1 Study population

Among the 65 patients [aged was 66 (25) years; 69% were men, and 93% Caucasian] treated in the hemodialysis unit of the General University Hospital in Prague, 29 patients met the inclusion criteria. The main reasons for non-inclusion were cognitive dysfunction, refusal to participate, inability to perform lung function testing (e.g., poor cooperation or physical limitations), and the presence of exclusion criteria such as acute cardiac or respiratory decompensation or intercurrent infection. Median the most frequent causes of ESKD were hypertensive kidney disease (31%) and multiple myeloma (14%) as far as there is a dedicated center for the treatment of this disease in our hospital. The dialysis vintage was 29 (45.5) months, and the time interval since the last hemodialysis session was 44 (21) h. All patients underwent hemodialysis three times a week. Ultrafiltration during the hemodialysis session was 2,000 (1,000) mL and residual diuresis was 500 (800) mL/24 h. Hemodialysis was performed via arteriovenous access in 19 patients (67%), and 10 patients (33%) were dialyzed through a catheter. Chronic cardiac failure was diagnosed in 5 patients (17%), and 5 patients (17%) had chronic obstructive pulmonary disease. Sixteen patients (55%) had a history of COVID-19 infection. Mild dyspnea (NYHA I-II) was present in 20 patients (69%), and the remaining 9 patients (31%) were in NYHA class III. After hemodialysis, 8 patients (28%) experienced fatigue, 8 patients (28%) felt weak, and 3 patients (10%) reported headache. Echocardiographic parameters are presented in Table 1, and other baseline data are shown in Tables 2–4.

**Table 1:** Baseline echocardiographic data of analyzed patients before hemodialysis.

Echocardiographic parameter	Value
Ejection fraction of the left ventricle (%)	60.1 ± 10.9
Cardiac index (L/min/m^2^)	3.2 (1.9)
Left ventricular end-diastolic volume indexed to BSA (mL/m^2^)	64 ± 19
Left ventricular mass indexed to BSA (g/m^2^)	118 ± 31
Left atrial volume indexed to BSA (mL/m^2^)	40 (14)
Fractional area change of the right ventricle (%)	42 ± 12
End-diastolic area of the right ventricle (cm^2^)	20 ± 5
End-systolic area of the right ventricle (cm^2^)	11 ± 5
E/e' average	9.1 (6.7)
E/A	0.84 ± 0.36
Estimated pulmonary arterial systolic pressure (mmHg)	28.8 ± 14.7

Data are reported as the mean ± standard deviation for variables with a Gaussian distribution or as the median (interquartile range) for variables with a non-Gaussian distribution. BSA, body surface area, E/e', ratio between early mitral inflow velocity and mitral annular early diastolic velocity, E/A, early to late diastolic transmitral flow velocity, PASP, estimated pulmonary arterial systolic pressure.

**Table 2:** Lung function changes after hemodialysis session.

Pulmonary function parameter	Before HD	After HD	*p*-value	Z
Vital capacity (L)	3.3 ± 1.14	3.35 ± 1.08	0.314	
Vital capacity (% of predicted value)	84.41 ± 17.68	87.04 ± 15.51	0.09	
Total lung capacity (L)	**5.11** **±1.18**	**5.34** **±1.27**	**0.005**	
Total lung capacity (% of predicted value)	**80.19** **±13.17**	**83.15** **±13.2**	**0.027**	
Forced expiratory volume in 1 s (L)	**2.44** **±0.88**	**2.54** **±0.9**	**0.022**	
Forced expiratory volume in 1 s (% of predicted value)	80.19 ± 16.19	83.26 ± 15.7	0.71	
FEV1/FVC (% of predicted value)	98.89 ± 8.46	100.86 ± 8.33	0.081	
Diffusing capacity of the lung for carbon monoxide (ml/min/mmHg)	**14.48 (4.35)**	**15.73 (4.78)**	**0.0014**	**3.19**

Data are reported as mean ± standard deviation in variables with Gaussian distribution or as median (quartile range) in variables with non-Gaussian distribution. Bold values denote statistically significant results (*p* < 0.05).

**Table 3:** Body composition changes after hemodialysis session.

Body composition parameter	Before HD	After HD	*p*-value	Z
Overhydration (L)	**1.8 (1.35)**	**0.4 (1.9)**	**< 0.0001**	**4.28**
Total body water (L)	**37.45** **±8.51**	**35.58** **±8.53**	**0.0009**	
Extracellular water (L)	**18.35** **±4.15**	**16.57** **±4**	**< 0.0001**	
Intracellular water (L)	19.19 ± 4.58	19 ± 4.84	0.528	
Relative hydration index	**0.1 (0.06)**	**0.02 (0.14)**	**< 0.0001**	**4.16**

Data are reported as mean ± standard deviation in variables with Gaussian distribution or as median (quartile range) in variables with non-Gaussian distribution. Bold values denote statistically significant results (*p* < 0.05).

**Table 4:** Correlations between changes in pulmonary function parameters and ultrafiltration.

Pulmonary function parameter	R	*P*-value
Vital capacity (L)	0.32	0.1
Vital capacity (% of predicted value)	0.2	0.31
Total lung capacity (L)	−0.11	0.59
Total lung capacity (% of predicted value)	−0.21	0.3
Forced expiratory volume in 1 s (L)	0.22	0.27
Forced expiratory volume in 1 s (% of predicted value)	0.21	0.3
FEV1/FVC (% of predicted value)	0.17	0.38

FEV1/ FVC, Forced expiratory volume in 1 s to forced vital capacity ratio.

There was a significant increase in TLC and FEV1 after hemodialysis. DLCO increased significantly after hemodialysis (see Table 2 for details). Patients with chronic lung disease had significantly lower changes in the FEV1/FVC after hemodialysis (2(5) vs. −2(6); *p* = 0.033) and had significantly higher partial pressure of CO2 after hemodialysis (0.13(0.41) vs. 0.64(0.52); *p* = 0.024). Changes in other lung function parameters, dynamometry, body composition, and ion and blood gas levels were not significantly different compared with patients without or with chronic lung disease, patients with chronic cardiac failure, or those with a history of COVID-19 infection.

As expected, hydration was significantly lower after hemodialysis. In the bioimpedance analysis, this difference was attributable to a decrease in extracellular water (ECW) volume, as the volume of intracellular water (ICW) did not change significantly within 1 h after dialysis (see Table 3 for details). DLCO change in percents correlated with ultrafiltration (-0.43, p=0.022), decreases in body weight and overhydration (R= −0.42, *p* = 0.02; R= −0.55, *p* = 0.003, respectively). No significant correlations were observed between ultrafiltration and pulmonary function parameters, including lung volumes and airflow indices obtained during lung function testing, details are shown in Table 4.

Muscle strength measured by dynamometry was significantly lower after hemodialysis (25.31 ± 12.82 vs. 23.79 ± 12.07; *p* = 0.008). However, no significant relationships were observed between changes in muscle strength and the results of pulmonary function tests or bioimpedance data.

Changes in electrolyte and blood gas after hemodialysis are presented in Table 5.

**Table 5:** Electrolyte and blood gas changes after hemodialysis session.

Laboratory parameter	Before HD	After HD	*p*-value
Natrium (mmol/L)	138.9 ± 2.74	138.7 ± 1.79	0.62
Potassium (mmol/L)	**5.29** **±0.99**	**4.38** **±0.38**	**< 0.0001**
Chloride (mmol/L)	102.31 ± 4.07	101.31 ± 2.29	0.15
Calcium (mmol/L)	**2.25** **±0.17**	**2.49** **±0.18**	**< 0.0001**
Magnesium (mmol/L)	**0.96** **±0.16**	**0.84** **±0.08**	**< 0.0001**
Albumin (g/L)	**38.18** **±5.87**	**41.24** **±7.65**	**0.0004**
Total blood protein (g/L)	**64.18** **±9.14**	**69.11** **±10.76**	**0.0004**
Leukocytes (10^9^/L)	7.61 ± 2.71	7.22 ± 2.97	0.112
Red blood cells (10^12^/L)	**3.42** **±0.63**	**3.72** **±0.66**	**< 0.0001**
Hemoglobin (g/L)	**103.83** **±17.81**	**112.93** **±18.41**	**< 0.0001**
Hematocrit	**32.63** **±6.26**	**34.86** **±6.3**	**< 0.0001**
Thrombocytes (10^9^/L)	224.0 ± 79.47	227.62 ± 100.12	0.692
pH	**7.34** **±0.05**	**7.42** **±0.04**	**< 0.0001**
pCO_2_ (mmHg)	**39.98** **±5,18**	**41.55** **±4.5**	**0.027**
HCO_3_ actual (mmol/L)	**20.89** **±2.59**	**26.5** **±1.15**	**< 0.0001**
pO_2_ (mmHg)	82.51 ± 32,63	82.13 ± 37.35	0.9
SpO_2_ (%)	86.67 ± 14.86	83.9 ± 19.55	0.231

Data are reported as mean ± standard deviation in variables with Gaussian distribution or as median (quartile range) in variables with non-Gaussian distribution. pCO_2_, Partial pressure of carbon dioxide; HCO_3_, Bicarbonate; pO_2_, Partial pressure of oxygen; SpO_2_, Oxygen saturation. Bold values denote statistically significant results (*p* < 0.05).

### 3.2 Correlation analysis

Total Body Water (TBW) and ICW before dialysis were associated with residual diuresis (R = 0.41, *p* = 0.036 and R = 0.44, *p* = 0.023, respectively). Patients with higher left ventricular filling pressure had significantly lower VC, FVC and FEV1 before hemodialysis (see Table 6 for details).

**Table 6:** Correlations of lung function with diastolic filling of the left ventricle.

Correlated parameters	R	*p* value
VC before hemodialysis (L) to E/e' avg.	**−0.63**	**0.006**
VC before hemodialysis (L) to E/A	**−0.54**	**0.014**
FVC before hemodialysis (L) to E/e' avg.	**−0.68**	**0.003**
FVC before hemodialysis (L) to E/A	**−0.57**	**0.011**
FEV1 before hemodialysis (L) to E/e' avg.	**−0.712**	**0.001**
FEV1 before hemodialysis (L) to E/A	**−0.59**	**0.006**

VC, vital capacity of the lungs; FVC, forced vital capacity; FEV1, forced expiratory volume in the 1 s. Bold values denote statistically significant results (*p* < 0.05).

The statistically significant relationship between lung volume and BIA indicated an inverse correlation between the change in TLC percentage and the change in TBW (R = 0.45, *p* = 0.025) and between the change in TLC percentage and residual diuresis (R = 0.6, *p* = 0.001). Additionally, the change in FVC correlated with serum calcium levels (R = −0.49; *p* = 0.009).

Changes in DLCO were associated with an increase in heart rate (R = 0.56, *p* = 0.04). The increase in TLC in liters was related to the change in leukocyte count (R = 0.46; *p* = 0.017). The Tiffeneau index (FEV1/FVC) was inversely related to serum calcium levels (R = −0.49; *p* = 0.009).

## 4 Discussion

Significant improvements in lung volume, airway flow and alveolar–capillary membrane function were observed after a single hemodialysis session. Despite weakness, fatigue and headache shortly after hemodialysis, 13% of patients experienced improvement in dyspnea. These improvements were associated with changes in body hydration and laboratory test results. The correlation of ventilation parameters with left ventricular filling pressure highlights the interaction between cardiac function, especially cardiac failure, and respiratory mechanics patients with ESKD. Surprisingly, there was no significant relationship between pulmonary function and muscle strength. Prior to hemodialysis, most patients did not meet the criteria for obstructive or restrictive lung disease, although DLCO values were slightly reduced. Fluid overload and laboratory findings, such as mild hyperkalemia and elevated urea and creatinine levels, were consistent with expected ESKD-related abnormalities. Residual diuresis correlated positively with TBW and ICW before hemodialysis. The underlying mechanisms may include more effective regulation of volume and osmotic homeostasis, but further research is necessary.

The lack of correlations between lung volumes, flows and ultrafiltration suggests that the observed improvements are not solely dependent on the absolute amount of fluid removed. Hemodialysis induces not only fluid removal, but also redistribution of body fluids, including a reduction in pulmonary interstitial and vascular congestion, which may not be directly proportional to ultrafiltration volume. In addition, changes in acid–base balance, electrolyte composition, and uremic toxin clearance may contribute to improved respiratory mechanics and gas exchange. At the same time, we observed a significant inverse correlation between changes in TBW and TLC. Ultrafiltration represents the prescribed volume of fluid removal, whereas bioimpedance-derived TBW reflects the actual amount of body fluids across compartments. The relationship between TBW and lung volumes therefore likely captures changes in pulmonary interstitial and vascular congestion more accurately than ultrafiltration volume alone.

The increase in TLS is most likely related to improved lung aeration and reduction in pulmonary congestion following fluid removal. Although changes in surfactant function could theoretically contribute (24), this mechanism was not assessed in the present study and therefore remains speculative. Consistent with the study of Navari et al., we observed a significant increase in FEV1 after hemodialysis (23, 25). Rahgoshai et al. reported an improvement in FVC after hemodialysis but no change in FEV1, VC, or the FEV1/VC ratio. However, Navari et al. studied a younger population, and in the studies of both Navari and Rahgoshai, acetate and bicarbonate hemodialysis were used; however, only forced volumes and flows were measured, and no data concerning ultrafiltration or body composition were recorded (26, 27). Reduced airflow before dialysis has been attributed to pulmonary edema or bronchial congestion with mucosal edema (28–30). The smaller improvement in FEV1/FVC observed in patients with chronic lung disease suggests that the respiratory benefits of hemodialysis may be greater in individuals whose dyspnea is driven primarily by fluid overload and pulmonary congestion rather than fixed airway obstruction. This finding highlights the potential importance of underlying pulmonary pathology in modulating the response to fluid removal. Future studies should prospectively evaluate respiratory outcomes stratified by pulmonary diagnosis.

As expected, DLCO increased significantly after hemodialysis. This improvement correlated with ultrafiltration, increased heart rate and OH/ECW ratio, suggesting that an improved alveolar–capillary surface area, together with increased heart rate, may contribute to improved gas transfer. Similar findings were reported by Herrero et al., who observed decreased DLCO in patients undergoing long-term hemodialysis compared with those with CKD and those with preserved renal function, although respiratory mechanics were unaffected (31).

Even though muscle strength decreased significantly after hemodialysis, which was consistent with prior studies (32), this did not appear to influence pulmonary function. One possible explanation is that the relief of pulmonary congestion is a substantially stronger mechanism than the decline in muscular strength. Hematological changes after dialysis followed the expected patterns: hematocrit and erythrocyte count increased because of hemoconcentration, whereas leukocyte and thrombocyte counts did not change significantly. Variations in leukocyte counts are influenced by activation, redistribution between circulating and marginating pools, and adherence to the dialysis membrane. Platelet changes are similarly modulated by complement activation and increased blood viscosity (33, 34).

The observed associations between changes in pulmonary function parameters and leukocyte count may reflect the complex inflammatory and hemodynamic responses during hemodialysis. Leukocyte dynamics during dialysis are influenced by activation, transient sequestration in the pulmonary circulation, and subsequent redistribution, which may affect pulmonary microcirculation and gas exchange (35, 36). Similarly, although platelet counts did not change significantly, platelet activation during hemodialysis may contribute to microvascular alterations, including within the pulmonary circulation (37, 38). These processes could potentially influence alveolar–capillary function; however, the clinical significance of these findings remains uncertain. We also observed a significant association between changes in pulmonary function parameters and serum calcium levels. Although the exact mechanism is unclear, calcium plays an important role in smooth muscle contraction, including bronchial tone, and may therefore influence airway dynamics. In addition, calcium levels may reflect broader metabolic and hormonal changes in patients with ESKD, including disturbances in mineral metabolism and acid–base balance, which could indirectly affect respiratory function (39).

The lack of direct association between muscle strength and pulmonary function indicates that respiratory improvements are driven primarily by hemodynamic and fluid shifts rather than by muscular performance. The positive correlation between residual diuresis and intracellular hydration indicates that preserved kidney function may help maintain cellular integrity and support a more balanced distribution of body water.

Overall, these results highlight the importance of precise fluid management and preservation of residual kidney function in optimizing respiratory outcomes and overall wellbeing in hemodialysis patients. Residual diuresis showed substantial variability in the study cohort and stratified analyses was not feasible due to the limited sample size, so this may represent an important source of unmeasured confounding. Future studies with larger cohorts and longitudinal designs are needed to confirm these findings and to explore the long-term consequences of hydration dynamics on pulmonary and cardiovascular health in patients with ESKD.

## Data Availability

The raw data supporting the conclusions of this article will be made available by the authors, without undue reservation.
